# C-undecylcalix[4]resorcinarene Langmuir–Blodgett/Porous Reduced Graphene Oxide Composite Film as a Electrochemical Sensor for the Determination of Tryptophan

**DOI:** 10.3390/bios13121024

**Published:** 2023-12-10

**Authors:** Yanju Wu, Keyu Chen, Fei Wang

**Affiliations:** School of Chemical Engineering and Dyeing Engineering, Henan University of Engineering, Zhengzhou 450007, China; yjwu2008@163.com (Y.W.); ywwang2012@163.com (K.C.)

**Keywords:** Langmuir–Blodgett film, porous reduced graphene oxide, calix[4]arene, electrochemical sensing, tryptophan

## Abstract

In this study, a composite film was developed for the electrochemical sensing of tryptophan (Trp). Porous reduced graphene oxide (PrGO) was utilized as the electron transfer layer, and a C-undecylcalix[4]resorcinarene Langmuir–Blodgett (CUCR-LB) film served as the molecular recognition layer. Atomic force microscopy (AFM), transmission electron microscopy (TEM), Raman spectroscopy, scanning electron microscopy (SEM), and electrochemical experiments were employed to analyze the characteristics of the CUCR-LB/PrGO composite film. The electrochemical behavior of Trp on the CUCR-LB/PrGO composite film was investigated, revealing a Trp linear response range of 1.0 × 10^−7^ to 3.0 × 10^−5^ mol L^−1^ and a detection limit of 3.0 × 10^−8^ mol L^−1^. Furthermore, the developed electroanalytical method successfully determined Trp content in an amino acid injection sample. This study not only introduces a rapid and reliable electrochemical method for the determination of Trp but also presents a new strategy for constructing high-performance electrochemical sensing platforms.

## 1. Introduction

Tryptophan (Trp), as a pivotal constituent among the eight essential amino acids, plays a key role in regulating body growth and metabolic processes [1]. Research has indicated that abnormal levels of Trp can potentially contribute to the development of some serious diseases, such as delusional disorders and Alzheimer’s disease [2]. Furthermore, the determination of Trp serves as a critical means for the early diagnosis of diseases. For example, the concentration of Trp in gastric fluid can yield valuable information about early-stage gastric cancer [3]. Therefore, it is crucial to develop a dependable and effective method for the determining of Trp in real samples.

Several methods, including fluorescence spectroscopy [4], capillary electrophoresis [5], and high-performance liquid chromatography (HPLC) [6], have been proposed to determinate trace amounts of Trp. Despite their widespread usage, these techniques entail certain limitations, such as long detective times, costly instrumentation, intricate analytical processes, and the use of toxic solvents [7]. Over the years, electrochemical sensing methods have emerged as a promising approach for the determination of bioactive molecules [8]. This is primarily attributed to their inherent advantages, including rapid response, easy miniaturization, high sensitivity, and low cost. On bare electrodes, however, the sensitivity for the determination of Trp is limited due to the sluggish electrode kinetics resulting from high overpotentials [9]. To address this challenge, the modification of electrodes, with enhanced sensitivity, has emerged as an effective solution. Various materials, including noble metals [10], carbon-based materials [11], functional polymers [12,13], and semiconductors [14], have been employed to modify electrodes and have successfully demonstrated the determination of Trp in diverse real-world samples. Nonetheless, these electrochemical sensors still suffer from limitations, such as narrow linear ranges, insufficient detection limits (LOD), and high costs associated with the use of noble metals [15]. Therefore, it is necessary to explore and develop more efficient electrochemical sensing for the determination of Trp.

Porous reduced graphene oxide (PrGO) has emerged as a superior material for electrochemical sensing [16]. It has all the advantages of reduced graphene oxide (rGO) for electrochemical sensing, such as facilitated electron transfer and increased active surface area. Furthermore, its unique structure can mitigate aggregation and stacking, thereby promoting mass and ion transmission [17,18]. Wang H.Y. demonstrated the notable advantages of PrGO in the electrochemical sensing of rifampicin [19]. Similarly, Rabah B et al. presented a simple and efficient strategy for immobilizing anti-gliadin polyclonal antibodies on PrGO-modified glassy carbon electrodes (GCE) [20]. The resulting immunosensor exhibited high sensitivity to gliadin due to the excellent electron transfer channels facilitated by the support of PrGO.

Calixarenes are cyclic molecules with a rigid structure that can selectively bind to specific analytes [21]. This binding interaction between the analyte and calixarene can be conveniently translated into an electrochemical signal, allowing for a highly selective and sensitive analysis of the samples. Siriboon M et al. reported the composite film of calix[6]arene/bismuth ferrite/multi-walled carbon nanotube for the electrochemical sensing of methyl parathion [22]. The results demonstrated that calix[6]arene can form a host–guest interaction with methyl parathion. Its introduction promotes the selective enrichment of methyl parathion on the surface of the electrode, which improves the sensitivity and selectivity of the sensor. On the other hand, the Langmuir–Blodgett (LB) technique enables the fabrication of uniform and ultra-thin films at the molecular level. By increasing the transferred surface pressure and the number of layers, it is possible to construct films modified with calixarene that are well arranged, tightly packed, and have a higher density of active centers. Hao Q L et al. proposed the modification of a pre-oxidized gold electrode with a mixed LB film of 4-tert-butylcalix[6]arene and cellulose acetate, which demonstrated excellent recognition and sensing capabilities towards dopamine (DA) [23]. Recently, our research group introduced a pre-treated GCE modified with C-undecylcalix[4]resorcinarene LB (CUCR-LB) film [24]. It was utilized as an electrochemical sensing platform for the simultaneous detection of dopamine (DA) and uric acid (UA). The synergistic effect of the CUCR-LB film and the pre-treated GCE resulted in high sensitivity and selectivity.

**Scheme 1:**
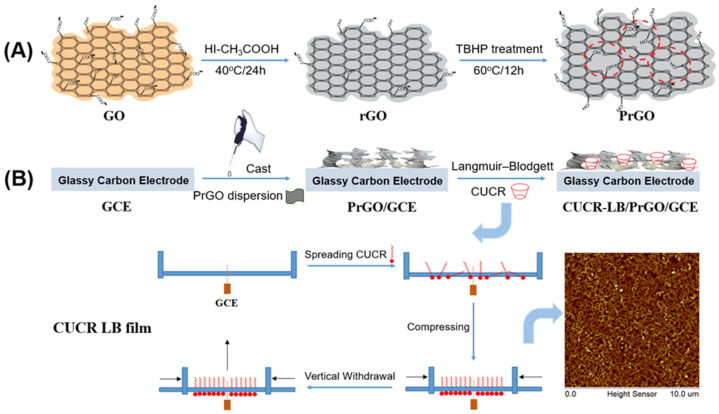
Schematic procedure for the fabrication of PrGO (**A**) and CUCR-LB/PrGO/GCE (**B**).

Herein, a novel electrochemical sensing platform was presented to determinate Trp based on a composite film of CUCR-LB film and PrGO-modified GCE (CUCR-LB/PrGO/GCE). Scheme 1 shows the fabrication process of PrGO and CUCR-LB/PrGO/GCE. PrGO serves as an electron transfer layer, which helps to enhance the electron transfer between Trp molecules and the surface of the electrode. On the other hand, CUCR-LB acts as a molecular recognition layer, which increases the adsorption capacity of Trp molecules on the surface of the electrode. Through the combination of the advantages of PrGO and CUCR-LB film, the composite film exhibits enhanced electrochemical response towards Trp, surpassing that of the pure PrGO film. This novel sensing platform holds great potential for various applications in the determination of Trp.

## 2. Materials and Methods

### 2.1. Materials

C-undecylcalix[4]resorcinarene (CUCR), tryptophan (Trp), tert-butanol hydrogen peroxide (TBHP), graphite powder, potassium, permanganate (KMnO_4_), and other chemical reagents were obtained from Shanghai Aladdin Chemical Reagent Co., LTD. (Shanghai, China). All the reagents were of analytical grade and were used without any additional purification.

### 2.2. Instruments

Atomic Force Microscopy (AFM) images were obtained by a Bruker of Dimension FastScan atomic force microscopy (Bruker Co., Germany), and the data were analyzed using Namoscope analysis 1.5. UV-Vis absorbance spectra were recorded on a UV-2102 spectrophotometer (Unico Co., Shanghai, China) using UVwin 5.1.0 control software. Raman spectra were conducted using an inVia Reflex Raman spectrometer (Renishaw Co., UK). Transmission electron microscopic (TEM) images were acquired using a JEM2100 transmission electron microscope (Japan Electronics Co., Japan). Scanning electron microscopic (SEM) images were obtained using a Quonxe-250 scanning electron microscope (FEI Co., Czech). Electrochemical tests were performed on a CHI 660E electrochemical analyzer (Shanghai Chenhua Instrument Co., Shanghai, China) using a conventional three-electrode system.

### 2.3. Preparation of Porous Reduced Graphene Oxide

Graphene oxide (GO) was prepared using a previously established method [25]. Subsequently, rGO was obtained by reducing GO with hydrazine [26]. To obtain PrGO, 100 mg of rGO was dispersed in a 100 mL TBHP solution (100 mL) and sonicated for 0.5 h. The resulting mixture was then refluxed at 60 °C for 12 h. After filtration, the collected PrGO powder was subjected to dialysis to remove any residual TBHP. Finally, the PrGO powder was obtained through freeze-drying.

### 2.4. Fabrication of the Composite Film-Modified Electrode

A GCE (*d* = 3 mm) was polished by a conventional method. A total of 0.5 mg mL^−1^ PrGO was dispersed in N-methylpyrrolidone and then sonicated in an ice bath for 1 h to ensure thorough mixing and dispersion. An 8-μL solution of the PrGO dispersion was then carefully deposited onto the tip of the electrode. The modified electrode was then dried at a temperature of 60 °C for 1 h. It was named PrGO/GCE. Similarly, an rGO-modified electrode was prepared (rGO/GCE). Subsequently, a CUCR-LB film was fabricated and transferred onto the PrGO/GCE under a surface pressure of 30.0 mN m^−1^. This modified electrode was designated as CUCR-LB/PrGO/GCE. Multilayer films were assembled by sequentially transferring monolayers.

## 3. Results and Discussion

### 3.1. Characteristics of CUCR-LB Film

**Figure 1:**
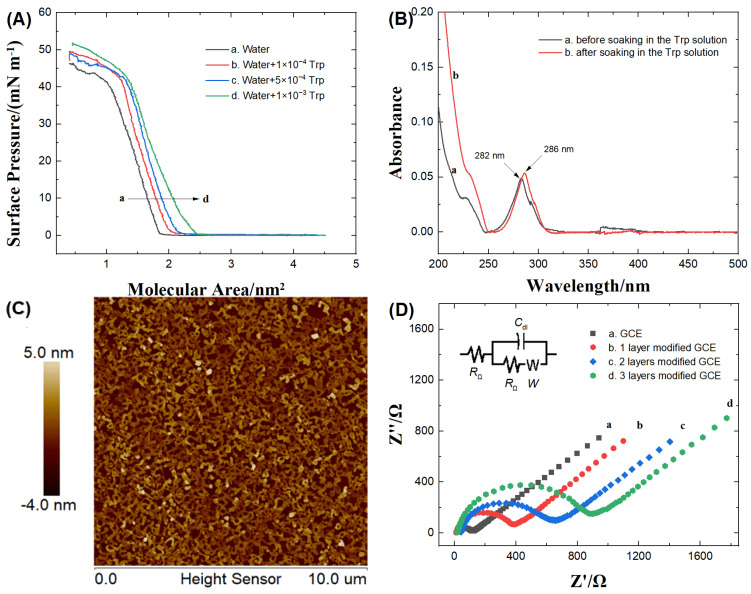
(**A**) π~A isotherms of CUCR monolayer on pure water and aqueous subphase with different Trp concentrations; (**B**) UV-vis spectral curves of 20 layers of CUCR-LB film on quartz substrate before and after soaking in a 1.0 × 10^−4^ mol L^−1^ Trp solution for 30 min; (**C**) AFM 2D morphology images of mica-covered CUCR-LB film under 30 mN m^−1^ transfer surface pressures (scan area: 10 μm × 10 μm); (**D**) Nyquist plots of bare GCE and different layers of CUCR-LB film-modified GCE in a 5 × 10^−3^ mol L^−1^ [Fe(CN)_6_]^3−/4−^ + 0.2 mol L^−1^ KCl solution, with an insert showing the equivalent circuit.

According to our previous results, CUCR is able to form a stable monolayer at the gas–liquid interface [24]. Figure 1A displays the isotherms of surface pressure (π)~molecular area (A) for CUCR on pure water and aqueous subphase with varying concentrations of Trp. In the case of CUCR monolayers formed on pure water, the surface pressure remained low at relatively high molecular areas. As compression increased, the isotherms exhibited a sharp rise in surface pressure. A collapse of the CUCR monolayer occurred when the surface pressure surpassed 48 mN m^−1^. When the linear portion of the isotherm was extrapolated to π = 0, the mean area of CUCR in the condensed state was determined to be 1.85 nm^2^. This result was comparable to the reported mean area of calix[4]arene [27]. As the concentration of Trp in the subphase aqueous solution increased, the isotherm shifted rightward, and the mean area of CUCR increased. This indicates a binding interaction between CUCR and Trp molecules. To further validate this result, UV-visible spectral curves were measured. Figure 1B shows the UV-visible spectral curve of a 20-layer CUCR-LB-modified film deposited on a quartz sheet before and after soaking in a solution containing 1.0 × 10^−4^ mol L^−1^ Trp for 30 min. It was evident that the CUCR-LB film displayed a distinctive absorption peak at 282.0 nm, resulting from the presence of the phenyl group [28]. Upon immersion of the modified quartz in the Trp solution, the absorption peak underwent significant enhancement and shifts to 286.0 nm. These spectral changes once again confirm the interaction between the CUCR-LB film and Trp molecules, indicating its ability to effectively enrich Trp molecules.

Atomic Force Microscopy (AFM) was used to study the morphology of CUCR-LB films. Figure 1C presents the AFM image of the CUCR-LB film on the mica surface. It was evident that a dense, ordered, and ultra-thin film with regularly distributed small pores could be observed. The film surface appeared relatively flat, and there was no significant molecular aggregation, indicating the good stability and reproducibility of the film. To further characterize the CUCR-LB films, electrochemical impedance spectroscopy (EIS) was utilized. Figure 1D displays the Nyquist plots of the bare GCE and the CUCR-LB-modified electrodes with varying layers in a 0.1 mol L^−1^ KCl solution containing a 5.0 × 10^−3^ mol L^−1^ K_3_[Fe(CN)_6_]/K_4_[Fe(CN)_6_] (1:1) solution. It can be observed that the Nyquist plot of the bare GCE showed a small semicircle diameter, indicating the easy accessibility of [Fe(CN)_6_]^4−/3−^ to the electrode surface and effective electron transfer [29]. For the CUCR-LB modified electrodes, the diameter of the semicircle in the Nyquist plots increased gradually with each additional layer. This could be attributed to the increasing density of the non-conductive CUCR-LB films with more layers. Using the equivalent circuit shown in the inset of Figure 1D, the relevant parameters were calculated and are listed in Table 1. There was a relationship between the double-layer capacitance (*C*_dl_), the thickness of films (*d*), and the number of layers (*N*): *C*_dl_ = (*ε*_calix_*ε*_0_*S*/*Nd*) [30]. Here, *ε*_0_ represents the vacuum permittivity with a magnitude of 8.85 × 10^−14^ F cm^−1^, and *ε*_calix_ corresponds to the relative dielectric constant of calixarene with a magnitude of 2.80 [30]. Through calculation, the thickness of one layer, two layers, and three layers of the CUCR-LB films were determined to be 4.4 nm, 9.0 nm, and 13.3 nm, respectively. A linear relationship between the number of layers and the film thickness of the CUCR-LB films was found, and these further emphasized the flatness and uniformity of the CUCR-LB films.

### 3.2. Characteristics of CUCR-LB/PrGO/GCE

**Figure 2:**
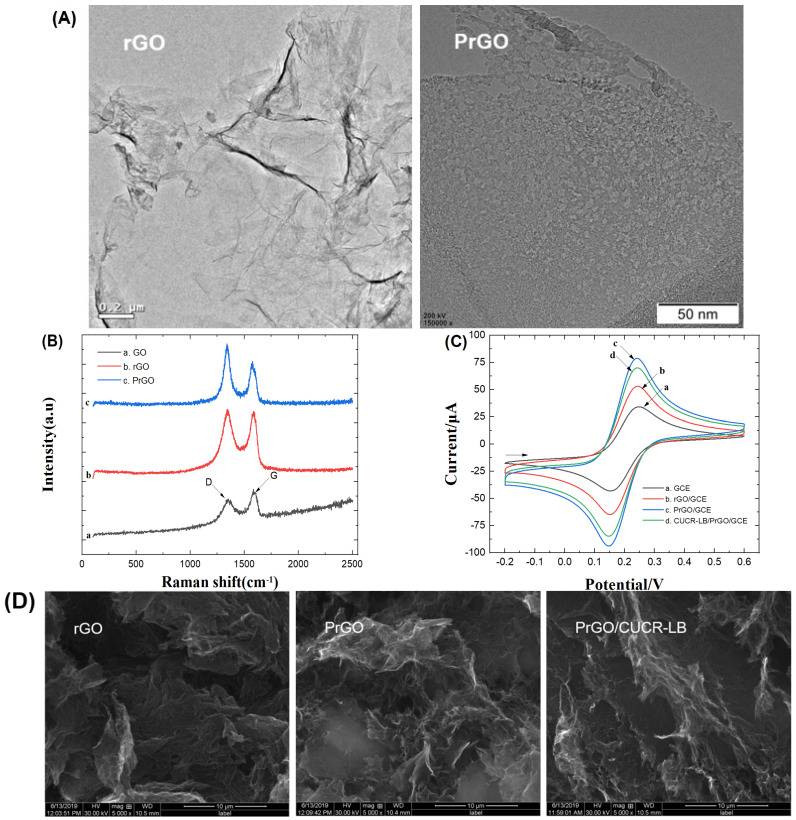
(**A**) TEM images of rGO and PrGO; (**B**) Raman spectra of GO, rGO, and PrGO; (**C**) CV curves of bare GCE, rGO/GCE, PrGO/GCE and CUCR-LB/PrGO/GCE in 1.0 × 10^−3^ mol L^−1^ K_3_[Fe(CN)_6_] + 0.1 mol L^−1^ KCl solution at a scan rate of 50 mV s^−1^; (**D**) SEM images of rGO, PrGO, and CUCR-LB/PrGO.

The transmission electron microscope (TEM) images of the prepared rGO and PrGO are shown in Figure 2A. In the rGO image, a transparent and continuous nanosheet structure was observed. After treatment with H_2_O_2_, uniformly distributed nanoscale pores could be clearly observed, with diameters ranging from approximately 5 to 10 nanometers. Furthermore, a Raman spectroscopic technique was used to characterize rGO and PrGO. According to Figure 2B, GO, rGO, and PrGO all exhibit two prominent characteristic peaks: a D peak at ~1345 cm^−1^ and a G peak at ~1590 cm^−1^ [31]. The integrity and ordering of the carbon lattice structure is reflected by the intensity ratio of the D peak to the G peak (*I*_D_/*I*_G_). Based on Raman testing, the *I*_D_/*I*_G_ ratio for GO, rGO, and PrGO was determined to be 0.86, 1.06, and 1.46, respectively. PrGO has a larger *I*_D_/*I*_G_ ratio, indicating a higher density of exposed edges and defects [32]. This situation was advantageous for enhancing the electron transfer.

To compare the characteristics of the different electrodes, cyclic voltammetry (CV) responses of various electrodes to a K_3_[Fe(CN)_6_] solution were examined (Figure 2C). Compared with the bare GCE electrode, the K_3_[Fe(CN)_6_] redox peaks of rGO/GCE, PrGO/GCE, and CUCR-LB/PrGO/GCE electrodes showed a significant increase in the redox peaks and a decrease in the peak-to-peak distance (Δ*E*_p_), indicating that these modified films had excellent electron transfer. Notably, the PrGO/GCE electrode showed the highest response peaks and the smallest Δ*E*_p_, suggesting that PrGO has better potential for electrochemical sensing applications compared to rGO. Compared with PrGO/GCE, the redox peaks of K_3_[Fe(CN)_6_] on the CUCR-LB/PrGO/GCE did not decrease significantly, indicating that the introduced CUCR-LB had no significant effect on the conductivity of the composite films. The electrochemical active surface area was calculated to be 0.052 cm^2^ for the bare GCE, 0.077 cm^2^ for rGO/GCE, 0.115 cm^2^ for PrGO/GCE, and 0.104 cm^2^ for CUCR-LB/PrGO/GCE according to the literature [33]. A higher electrochemical active surface area was expected to benefit the electrochemical sensing performance of Trp. Meanwhile, the morphology of the different modified electrodes was analyzed using scanning electron microscopy (SEM), as depicted in Figure 2D. On the rGO film, clusters of dense and wrinkled thin flakes were observed, exhibiting a pronounced aggregation phenomenon. On the PrGO film, a highly porous and interconnected network-like layered structure was observed, uniformly distributed on the surface of the electrode. It is pleasing to note that the aggregation phenomenon was significantly reduced. On the CUCR-LB/PrGO composite film, a similar network-like layered structure was observed, but the edges of the layers appeared slightly blurred, which was attributed to the non-conductive nature of CUCR-LB.

### 3.3. Electrochemical Behavior of Trp on CUCR-LB/PrGO/GCE

**Figure 3:**
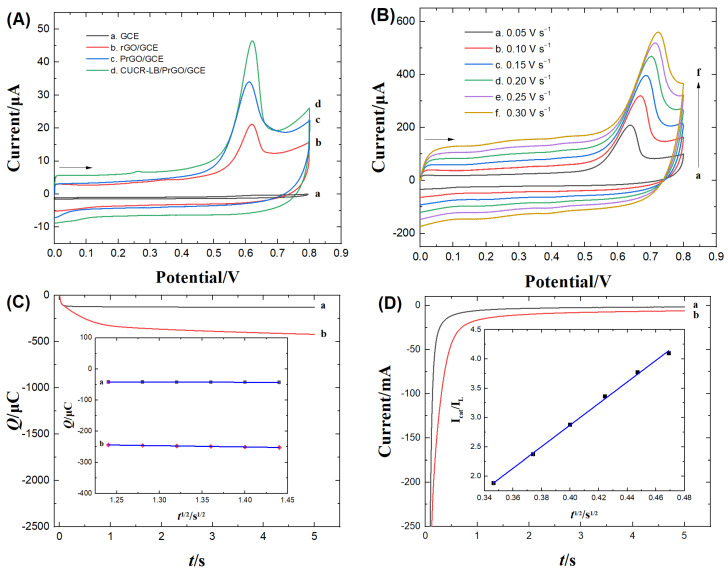
(**A**) CV curves of 2.0 × 10^−5^ mol L^−1^ Trp on a bare GCE, rGO/GCE, PrGO/GCE, and CUCR-LB/PrGO/GCE in a 0.2 mol L^−1^ phosphate-buffered solution (pH = 7.0) at a scan rate of 50 mV s^−1^; (**B**) CV curves of 2.0 × 10^−5^ mol L^−1^ Trp on the CUCR-LB/PrGO/GCE at different scan rates; (**C**) Chronocoulometric curves of absence (curve a) and presence (curve b) of 5.0 × 10^−4^ mol L^−1^ Trp on the CUCR-LB/PrGO/GCE, with the inset showing the corresponding relationship of *Q* versus *t*^1/2^; (**D**) Chronoamperometric curves of absence (curve a) and presence (curve b) of 5.0 × 10^−4^ mol L^−1^ Trp on the CUCR-LB/PrGO/GCE, with the inset showing the corresponding relationship of *i*_cat_/*i*_L_ versus *t*^1/2^.

The electrochemical response of various electrodes (including bare GCE, rGO/GCE, PrGO/GCE, and CUCR-LB/PrGO/GCE) to Trp was characterized by the CV method. The corresponding voltammograms are presented in Figure 3A, and the electrochemical data are listed in Table 2. A weak oxidation peak was observed on the bare GCE, which was due to the slow kinetics of the electrode caused by high overpotential. However, after modification with rGO and PrGO, the oxidation peak current (*i*_pa_) of Trp increased significantly due to the improved conductivity and electrochemically active surface area of rGO and PrGO. Notably, the CUCR-LB/PrGO/GCE exhibited the highest *i*_pa_ for Trp, being 50.9 times, 3.0 times, and 1.7 times higher than the bare GCE, rGO/GCE, and PrGO/GCE, respectively. This enhancement can be attributed to the synergistic effect between the PrGO and the CUCR-LB film. The PrGO film acted as the electron transfer layer, promoting the electron transfer rate of Trp, while the CUCR-LB film served as the recognition layer, enhancing the accumulation of Trp on the electrode surface. The combined effect of the PrGO and the CUCR-LB film resulted in a composite film that exhibited high sensitivity towards Trp.

The effect of the scan rate (*v*) on *i*_pa_ was investigated to study the electrochemical kinetics of Trp on the CUCR-LB/PrGO/GCE (Figure 3B). As the value of *v* increased, *i*_pa_ gradually increased, and the oxidation peak potential (*E*_pa_) shifted to a more positive potential. Furthermore, a linear relationship was observed between log*i*_pa_ and log*v* with a linear regression equation of log*i*_pa_ = −(3.139 ± 0.017), log*v* + (0.63 ± 0.02), and an R-value of 0.999. This suggests that the electrochemical oxidation of Trp on CUCR-LB/PrGO/GCE is under the control of both adsorption and diffusion [34].

Chronocoulometry (CC) and chronoamperometry (CA) were used to determine the saturated adsorption amount (Γ*) and catalytic rate constant (*k*_cat_) of Trp on various electrodes. The chronocoulometric curves of CUCR-LB/PrGO/GCE in blank and 5.0 × 10^−4^ mol L^−1^ Trp solutions and the corresponding *Q* vs. *t*^1/2^ linear plots are shown in Figure 3C. The Γ* of Trp on the CUCR-LB/PrGO/GCE was determined to be 1.17 × 10^−8^ mol cm^−2^ by calculating the difference in the intercept between the curves a and b [35]. Similarly, the calculated values of Γ* for Trp on other electrodes are listed in Table 2. Trp exhibited higher Γ* on the CUCR-LB/PrGO/GCE, indicating that the introduction of the CUCR-LB film indeed enhanced the loading of Trp on the surface of the electrode. Furthermore, the chronoamperometric curves of the CUCR-LB/PrGO/GCE in blank and 5.0 × 10^−4^ mol L^−1^ Trp solutions and the corresponding *i*_cat_/*i*_L_ vs. *t*^1/2^ linear plots are presented in Figure 3D. The *k*_cat_ of Trp on the CUCR-LB/PrGO/GCE was calculated to be 2.07 × 10^4^ mol L^−1^ s^−1^ using the slope of *i*_cat_/*i*_L_ vs. *t*^1/2^ [36]. Similarly, the calculated values of *k*_cat_ for Trp on other electrodes can be found in Table 2. These results indicate that Trp exhibited higher *k*_cat_ on the CUCR-LB/PrGO/GCE, suggesting that the synergistic effect of the PrGO film and the CUCR-LB film provided a more efficient interface for the electrochemical reaction of Trp.

### 3.4. Analytical Method Validations

#### 3.4.1. Optimization of Analysis and Detection Conditions

**Figure 4:**
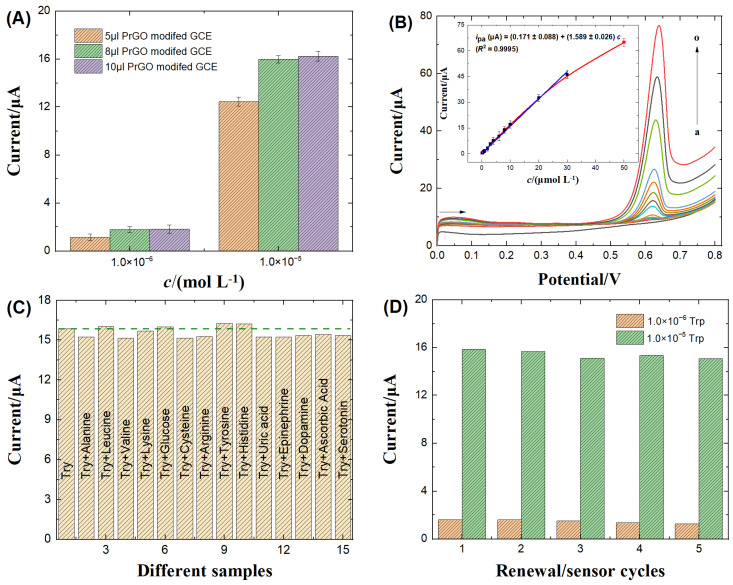
(**A**) Influence of varying amounts of PrGO on the oxidation currents of different concentrations of Trp detected at the CUCR-LB/PrGO/GCE; (**B**) LSV curves and corresponding calibration plot (the inset) for increasing concentrations of Trp on the CUCR-LB/PrGO/GCE (from *a* to *o*: 0.0, 0.1, 0.3, 0.5, 0.8, 1.0, 2.0, 3.0, 4.0, 6.0, 8.0, 10.0, 20.0, 30.0 and 50.0 (×10^−6^ mol L^−1^)); scan rate, 50 mV s^−1^; (**C**) Influence of different interfering compounds on the *i*_pa_ detected on the CUCR-LB/PrGO/GCE; (**D**) influence of renewal times on the oxidation currents of different concentrations of Trp detected on the CUCR-LB/PrGO/GCE.

To enhance sensitivity and minimize background current, Linear Sweep Voltammetry (LSV) was employed as the detection method in our analysis. Figure 4A demonstrates the influence of varying amounts of PrGO modification on the *i*_pa_ of Trp at concentrations of 1.0 × 10^−6^ mol L^−1^, and 1.0 × 10^−5^ mol L^−1^ within the CUCR-LB/PrGO/GCE electrochemical sensing system. The results indicate that the *i*_pa_ of Trp increased with escalating PrGO modification amount. However, increasing the PrGO modification amount from 8 μL to 10 μL did not result in a significant rise in current. Consequently, we selected an 8 μL PrGO modification for subsequent experiments to ensure an appropriate current response while minimizing excessive PrGO usage. Notably, the number of layers in the CUCR-LB film exhibited a contrary effect on the *i*_pa_ of Trp, where an increase led to a decrease. This phenomenon was attributed to the dense and non-conductive nature of CUCR-LB films, impeding electron transfer rates.

#### 3.4.2. Analytical Performances

An electroanalytical method for the determination of Trp using CUCR-LB/PrGO/GCE was established under optimized conditions. Figure 4B illustrates the linear sweep voltammograms of Trp at various concentrations. As the concentration of Trp increased, the *i*_pa_ gradually increased.

There was a strong linear relationship between the peak current values and the concentration in the range of 1.0 × 10^−7^ to 3.0 × 10^−5^ mol L^−1^ (the inset of Figure 4B), with a regression equation of *i*_pa_ (μA) = (0.171 ± 0.088) + (1.589 ± 0.026) *c* (μmol L^−1^) (*R* = 0.9995). Based on the signal-to-noise ratio of 3, the limit of detection (LOD) for Trp was determined to be 3.0 × 10^−8^ mol L^−1^ [37]. We compared the response performance of the CUCR-LB/PrGO composite film to other graphene-based composite films in terms of electrochemical sensing properties (Table 3). Except for SnS/TiO_2_@GO, nano-CeO_2_/rGO, CSOH/rGO, and CuO-CeO_2_-rGO-MWCNTs, the CUCR-LB/PrGO composite films have a wider linear range and better detection limits compared with other graphene-based composites. This indicates that using PrGO as the electron transfer layer and the CUCR-LB film as the recognition layer is a highly effective strategy for constructing an electrochemical sensing platform.

To evaluate the reproducibility of the CUCR-LB/PrGO/GCE for the detection of Trp, we conducted five parallel experiments. The relative standard deviation (*RSD*) was approximately 3.7%, demonstrating good reproducibility of the composite film for Trp detection. Furthermore, after storing the CUCR-LB/PrGO/GCE at 4 °C for 14 days, it still retained 92.2% of the response current for the concentration of 1.0 × 10^−5^ mol L^−1^ Trp, indicating the excellent stability of the CUCR-LB/PrGO/GCE.

#### 3.4.3. Interference Studies

The effect of potential interferents on the determination of Trp on the CUCR-LB/PrGO/GCE was investigated. The results showed that, even with 10-fold doses of interfering substances such as alanine, leucine, valine, lysine, glucose, cysteine, arginine, tyrosine, histidine, uric acid, epinephrine, dopamine, ascorbic acid, and serotonin, there were no significant effects on the peak current response and peak potential of Trp on the CUCR-LB/PrGO/GCE (Figure 4C). The results indicate that the CUCR-LB/PrGO composite film exhibits excellent selectivity in the electrochemical sensing of Trp.

#### 3.4.4. Electrode Renewal

The modified electrode was immersed in a 0.2 mol L^−1^ pH = 7.0 phosphate buffer (PB) solution and subjected to 5 min of stirring cleaning. Subsequently, CV was performed in the potential range of 0.0~0.8 V (vs. SCE) until the oxidation peak of Trp disappeared, achieving the renewal of the modified electrode. After five renewal/sensing cycles, the electrochemical response current for Trp at concentrations of 1.0 × 10^−6^ mol L^−1^ and 1.0 × 10^−5^ mol L^−1^ only decreased by 5.8% and 6.7%, respectively (Figure 4D). The performance of the renewed electrode was nearly identical to its initial state, indicating that the CUCR-LB/PrGO composite film was undamaged.

### 3.5. Determination of Trp in Amino Acid Injection Samples

The prospect of applying the established analytical method to analyze available amino acid injection samples is discussed. Amino acid injection samples were directly diluted 100 times directly with ultrapure water. An appropriate amount of the sample solution was determined using the standard addition method. The results showed no significant difference compared with the HPLC method (Table 4). The recoveries ranged from 98.7% to 104.0%, indicating that the CUCR-LB/PrGO composite films were highly reliable and practical.

## 4. Conclusions

In this work, a composite film for the electrochemical sensing of Trp was prepared using PrGO as the electron transfer layer and CUCR-LB as the molecular recognition layer. PrGO exhibited high electrochemical activity and accelerated electron transfer, while CUCR-LB film demonstrated strong recognition and enrichment capabilities for Trp molecules. Through the combination of the advantages of PrGO and CUCR-LB film, the composite film exhibited enhanced selectivity and sensitivity towards Trp. This not only provides a rapid and promising electrochemical method for the determination of Trp but also presents a new strategy for constructing high-performance electrochemical sensing platforms.

## Figures and Tables

**Table 1:** Relevant parameters of electrochemical impedance spectroscopy for a CUCR-LB film-modified electrode.

*N*	*C*_dl_ (F)	*W* (Ω cm^−2^)	*R*_Ω_(Ω)	*R*_ct_ (Ω)	*d* (nm)
1	3.967 × 10^−8^	0.003273	22.99	336.5	4.4
2	9.726 × 10^−9^	0.002009	32.32	540.6	9.0
3	4.388 × 10^−9^	0.001972	40.23	778.4	13.3

*C*_dl_: double-layer capacitance; *W*: Warburg impedance; *R*_Ω_: solution resistance; *R*_ct_: electron transfer resistance; *d*: thickness of films.

**Table 2:** Electrochemical reaction and kinetic characteristics of Trp on various electrodes.

Electrode	CV		CC and CA	
	*E*_pa_ (V)	*i*_pa_ (μA)	Γ* × 10^9^ (mol cm^−2^)	*k*_cat_ × 10^−3^ (mol L^−1^ s^−1^)
bare GCE	0.755	0.58	0.23	0.39
rGO/GCE	0.635	9.97	3.78	6.09
PrGO/GCE	0.621	17.89	9.17	10.9
CUCR-LB/PrGO/GCE	0.619	29.54	11.7	20.7

**Table 3:** Comparison of CUCR-LB/PrGO/GCE sensor for Trp detection with other graphene-based published modified electrodes.

Electrode	Methods	Linear Range (mol L^−1^)	Detection Limit (mol L^−1^)	Reference
TiO_2_-GR/4-ABSA/GCE	DPV	1.0 × 10^−6^~3.0 × 10^−5^	3.0 × 10^−7^	[9]
ErGO/GCE	DPV	2.0 × 10^−7^~4.0 × 10^−5^	1.0 × 10^−7^	[38]
AgNPs/P(Arg)-GO/GCE	DPV	1.0 × 10^−6^~1.5 × 10^−4^	1.22 × 10^−7^	[39]
Fe_3_O_4_@SiO_2_/GO/SPE	DPV	1.0 × 10^−6^~4.0 × 10^−4^	2.0 × 10^−7^	[40]
Ta_2_O_5_-rGO/GCE	LSV	1.0 × 10^−6^~8.0 × 10^−6^ 8.0 × 10^−6^~8.0 × 10^−5^ 8.0 × 10^−5^~8.0 × 10^−4^	8.4 × 10^−7^	[41]
SnS/TiO_2_@GO/GCE	DPV	1.33 × 10^−8^~1.57 × 10^−4^	7.8 × 10^−9^	[42]
(Au/Ag/Pd)NPs/EPGrO/GCE	DPV	1.0 × 10^−6^~6.0 × 10^−4^	3.0 × 10^−8^	[43]
rGO/AuNPs/SPE	DPV	5.0 × 10^−7^~5.0 × 10^−4^	3.9 × 10^−7^	[44]
nano-CeO_2_/rGO/GCE	LSV	1.0 × 10^−8^~1.0 × 10^−5^	6.0 × 10^−9^	[45]
CSOH/rGO/GCE	DPV	5.0 × 10^−8^~1.758 × 10^−5^	2.0 × 10^−9^	[46]
PDA/rGO-MnO_2_/GCE	DPV	1.0 × 10^−6^~3.0 × 10^−4^	2.2 × 10^−7^	[47]
CuO-CeO_2_-rGO-MWCNTs/GCE	DPV	1.0 × 10^−8^~1.35 × 10^−5^	7.3 × 10^−9^	[48]
Co_3_O_4_/rGO/GCE	LSV	1.0 × 10^−6^~8.0 × 10^−4^	2.6 × 10^−7^	[49]
CUCR-LB/PrGO/GCE	LSV	1.0 × 10^−7^~3.0 × 10^−5^	3.0 × 10^−8^	This work

TiO_2_-GR/4ABSA/GCE: TiO_2_-graphene/4-aminobenzenesulfonic acid composite film-modified glassy carbon electrode; AgNPs/P(Arg)-GO/GCE: Ag nanoparticles, graphene oxide- and poly(L-arginine)-modified glassy carbon electrode; Fe_3_O_4_@SiO_2_/GO/SPE: Fe_3_O_4_@SiO_2_/GO nanocomposite-modified graphite screen printed electrode; (Au/Ag/Pd)NPs/EPGrO/GCE: trimetallic nanoparticles of (Au/Ag/Pd)NPs uniformly capped electro-pretreated graphene oxide-modified glassy carbon electrode; CSOH/rGO/GCE: CuSn(OH)_6_ microsphere decorated on reduced graphene oxide-modified glassy carbon electrode; PDA/rGO-MnO_2_/GCE: polydopamine/reduced graphene oxide-MnO_2_-modified glassy carbon electrode; DPV: differential pulse voltammetry; LSV: linear sweep voltammetry; SPE: screen-printed electrode.

**Table 4:** Determination results of Trp in the amino acid injection samples using LSV and HPLC (*n* = 3).

Sample	LSV						HPLC
	Labeled (μmol L^−1^)	Detected (μmol L^−1^)	Added (μmol L^−1^)	Total Detected (μmol L^−1^)	RSD (%)	Recovery (%)	Detected (μmol L^−1^)
1 ^a^	5.00	5.10 (±0.14)	2.0	7.18 (±0.21)	2.9	104.0	5.08 (±0.05)
			20.0	24.83 (±0.30)	3.8	98.7	
2 ^b^	2.50	2.56 (±0.09)	2.0	4.61 (±0.13)	3.1	102.5	2.54 (±0.08)
			20.0	22.47 (±0.24)	3.9	99.6	

^a^. obtained from Guangzhou Green Cross Pharmaceutical Co., LTD. (Guangzhou, China). ^b^. obtained from Xuzhou Fifth Pharmaceutical Co., LTD. (Xuzhou, China).

## Data Availability

Data are contained within the article.
